# DNA Replication Control During *Drosophila* Development: Insights into the Onset of S Phase, Replication Initiation, and Fork Progression

**DOI:** 10.1534/genetics.115.186627

**Published:** 2017-08-31

**Authors:** Brian L. Hua, Terry L. Orr-Weaver

**Affiliations:** Whitehead Institute, Massachusetts Institute of Technology, Cambridge, Massachusetts 02142; Department of Biology, Massachusetts Institute of Technology, Cambridge, Massachusetts 02142

**Keywords:** FlyBook, *Drosophilamelanogaster*, origin activation, endocycle, differential replication, underreplication, gene amplification, rereplication

## Abstract

Proper control of DNA replication is critical to ensure genomic integrity during cell proliferation. In addition, differential regulation of the DNA replication program during development can change gene copy number to influence cell size and gene expression. *Drosophila melanogaster* serves as a powerful organism to study the developmental control of DNA replication in various cell cycle contexts in a variety of differentiated cell and tissue types. Additionally, *Drosophila* has provided several developmentally regulated replication models to dissect the molecular mechanisms that underlie replication-based copy number changes in the genome, which include differential underreplication and gene amplification. Here, we review key findings and our current understanding of the developmental control of DNA replication in the contexts of the archetypal replication program as well as of underreplication and differential gene amplification. We focus on the use of these latter two replication systems to delineate many of the molecular mechanisms that underlie the developmental control of replication initiation and fork elongation.

## DNA Replication Overview

Before cell division, the genome must be completely and accurately replicated to maintain the integrity of genetic information across cell generations. DNA replication initiates from thousands of DNA elements within the genome called origins of replication. Origins of replication direct the assembly of a large group of proteins and protein complexes to the site that ultimately allow for DNA unwinding and the establishment of two, bidirectional replication forks. DNA ahead of the fork is progressively unwound, generating single-stranded DNA that serves as a template for the synthesis of new DNA (Bleichert *et al.* 2017; Parker *et al.* 2017). Through the molecular study of DNA replication initiation and elongation, it is clear that the mechanisms that regulate origin activity and replication fork progression are diverse and complex, particularly in the context of development. *Drosophila* has provided powerful developmental systems to study both replication initiation and elongation at the cellular and molecular levels (Nordman and Orr-Weaver 2012). Here, we summarize important insights that the *Drosophila* system has shed upon the regulation of metazoan DNA replication. We then detail seminal studies that have led to critical understanding of the developmental control of replication origin activation and fork elongation. Finally, we address prevailing questions in DNA replication control and the outlook for the field.

## Protein Players at the Origin of Replication

**Figure 1:**
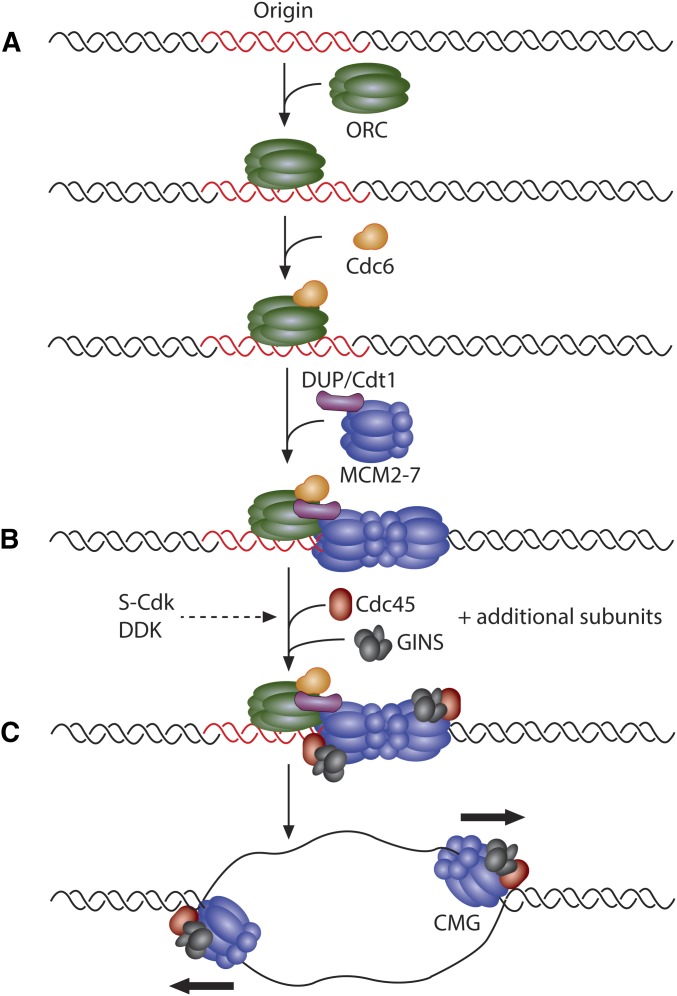
Stepwise assembly and activation of the CMG helicase. (A) An origin of replication is first recognized and bound by the origin recognition complex (ORC). Binding of ORC promotes the recruitment of the Cdc6 and DUP/Cdt1 initiation factors that work cooperatively to load the MCM2–7 helicase complex. (B) S-CDK and DDK activity are required for the subsequent recruitment of the helicase components Cdc45 and the GINS complex, along with additional subunits necessary for helicase function (Mus101/Dpb11, RecQ4/Sld2, and others). (C) The replicative helicase, composed of MCM2–7, Cdc45, and GINS, is activated at the start of S phase to begin replication.

DNA replication initiation requires the sequential recruitment and activation of a large number of replication protein components. Unlike in budding yeast, metazoan origins of replication are not defined by any known consensus sequence (Parker *et al.* 2017). However, protein factors required to establish the replication initiation complex and the replication fork are highly conserved in eukaryotes (Table 1). Replication initiation first requires that origins of replication are bound by the origin recognition complex (ORC) (composed of the six proteins ORC1–6) in late M and G1 phases of the cell cycle (Figure 1). The replication initiation factor cell division cycle 6 (Cdc6) is then recruited to the origin to form a complex with ORC. ORC and Cdc6 work cooperatively to recruit the initiation factor Cdt1 [Double Parked (DUP) in *Drosophila*] and the six-membered Minichromosome Maintenance (MCM)2–7 replicative helicase complex. In budding yeast, Cdt1 and MCM2–7 form a stable complex in cell lysates and are recruited to origins of replication together (Tanaka and Diffley 2002; Kawasaki *et al.* 2006; Remus *et al.* 2009). In *Xenopus* extracts, however, Cdt1 and MCM2–7 do not coprecipitate, suggesting that Cdt1 and the MCM2–7 complex may be recruited sequentially to replication origins in metazoans (Maiorano *et al.* 2000).

**Table 1:** Key proteins required for helicase loading and activation

*Drosophila*	Mammalian homolog	Budding yeast homolog	Function
ORC1	ORC1	Orc1	Helicase loading
ORC2	ORC2	Orc2	Helicase loading
Latheo	ORC3	Orc3	Helicase loading
ORC4	ORC4	Orc4	Helicase loading
ORC5	ORC5	Orc5	Helicase loading
ORC6	ORC6	Orc6	Helicase loading
CDC6	CDC6	Cdc6	Helicase loading
Double parked (DUP)	CDT1	Cdt1	Helicase loading
MCM2	MCM2	Mcm2	Helicase
MCM3	MCM3	Mcm3	Helicase
Disc proliferation abnormal (DPA)	MCM4	Mcm4	Helicase
MCM5	MCM5	Mcm5	Helicase
MCM6	MCM6	Mcm6	Helicase
MCM7	MCM7	Mcm7	Helicase
MCM10	MCM10	Mcm10	Helicase activation
CDC45	CDC45	Cdc45	Helicase activation/helicase
SLD5	SLD5	Sld5	Helicase activation/helicase
PSF1	PSF1	Psf1	Helicase activation/helicase
PSF2	PSF2	Psf2	Helicase activation/helicase
PSF3	PSF3	Psf3	Helicase activation/helicase
MUS101	TopBP1	Dpb11	Helicase activation/helicase
RECQ4	RECQL4	Sld2	Helicase activation
(Not identified)	Treslin/ticcr	Sld3	Helicase activation

Two hexamers of the MCM2–7 complex are loaded onto origin DNA in an inactive state before the onset of S phase. Under the regulation of two kinases, S phase Cyclin-Dependent Kinase (CDK) and Dbf4-Dependent Kinase (DDK), the MCM2–7 complex is joined by CDC45 and the Go-Ichi-Ni-San (GINS) complex, a four-membered complex composed of Sld5, Psf1, Psf2, and Psf3. Together, the CDC45/MCM2–7/GINS (CMG) complex forms the functional replicative helicase (Bleichert *et al.* 2017; Parker *et al.* 2017). As two MCM2–7 hexamers are loaded onto a single origin of replication, two CMG complexes establish the independent, bidirectional replication forks after origin activation (Figure 1).

## Hurdles for the Molecular Study of Metazoan DNA Replication

Despite the conservation of the proteins governing initiation of DNA replication in eukaryotes, there are complexities in the control of metazoan DNA replication. At the most fundamental level, it remains to be determined what dictates a replication origin and where ORC will bind in metazoans (Prioleau and MacAlpine 2016). This has limited analysis of the regulation of origin activation. In addition, initiation of replication within S phase is subject to more extensive regulation in metazoans than in budding yeast. Although in both only a subset of origins are activated at a given time point in S phase (Aparicio 2013), this effect becomes more pronounced during the prolonged period of S phase occurring in most metazoan cells. Furthermore, origins of replication are not uniformly distributed throughout the metazoan genome, resulting in large genomic regions that require the activity of replication forks emanating from distant origins for their replication (Debatisse *et al.* 2012). It also has been difficult to examine replication forks emanating from a single origin of replication. Finally, how developmental signals modulate the activity of replication origins and forks remains to be elucidated.

## Fundamentals of Drosophila DNA Replication and Insights Contributed to the DNA Replication Field

### Identification of replication proteins

Elegant genetic and biochemical studies initially performed in budding yeast allowed for a comprehensive identification of the key protein factors that are involved in origin activation and fork elongation (Bell and Labib 2016). Significantly, the minimal set of protein factors required for DNA replication in budding yeast *in vitro* has been described (Yeeles *et al.* 2015). The establishment of a cell-free replication system from *Xenopus* eggs allowed for powerful biochemical dissection of DNA replication in a metazoan system (Lohka and Masui 1983; Blow and Laskey 1986; Blow and Watson 1987; Hutchison *et al.* 1987; Almouzni and Mechali 1988). Seminal studies using this system led to the identification and functional characterization of several key replication factors in *Xenopus*, including the biochemical purification of an MCM-containing complex required for replication licensing (Chong *et al.* 1995) as well as the identification of *Xenopus* ORC2 and its essential role in replication initiation (Carpenter *et al.* 1996). Collectively, these studies played a significant role in demonstrating that yeast replication proteins are conserved in metazoans.

Whereas the budding yeast and *Xenopus* systems laid the groundwork in the identification of DNA replication factors and the molecular events that are required for replication initiation and fork elongation, *Drosophila* has since emerged as an extremely powerful organism to study metazoan DNA replication at both the molecular and developmental levels. For example, the metazoan homologs of the key replication initiation factor Cdt1 were first discovered in *Drosophila* (Whittaker *et al.* 2000) and *Xenopus* (Maiorano *et al.* 2000). Additionally, *Drosophila* mutants with impaired ORC2 and Cdt1 function showed gross defects in DNA replication, providing the first genetic evidence of the requirement of these conserved proteins in metazoans (Landis *et al.* 1997; Whittaker *et al.* 2000). Using biochemical methods, the functional helicase complex was shown to exist as a large protein assembly consisting of CDC45, MCM2–7, and GINS (CMG complex) through isolation from *Drosophila* embryo extracts (Moyer *et al.* 2006). Crucial structural insight into the regulation of metazoan DNA replication initiation resulted from extensive electron microscopy studies (Clarey *et al.* 2006, 2008) and the solving of the crystal structure of the *Drosophila* ORC complex (Bleichert *et al.* 2015). Finally, *Drosophila* has served as a metazoan model system to profile replication properties and dynamics genome-wide, beginning with the first genome-wide mapping of ORC in a differentiated metazoan cell type and tissue (MacAlpine *et al.* 2010; Sher *et al.* 2012). These genome-wide approaches have allowed for more comprehensive analysis of replication dynamics in the scope of the underlying chromatin landscape, developmental timing, and differentiation.

### Analysis of replication origins in Drosophila

Experiments using *Drosophila* cell culture lines have provided critical information about the timing of replication of genomic regions within S phase, localization of origins and sites of ORC binding, and the role of chromatin and histone modifications. Genome-wide techniques have allowed for comprehensive profiling of replication initiation sites in several *Drosophila* cell culture systems (Cayrou *et al.* 2011; Comoglio *et al.* 2015). Upon replication initiation, two nascent leading DNA strands extend from RNA primers located at the replication origin. These leading nascent strands can be isolated away from smaller RNA-primed Okazaki fragments on the lagging strand by size selection and from non-RNA-primed DNA by λ-exonuclease digestion (Gerbi and Bielinsky 1997). High-throughput sequencing of purified leading nascent strands then allows for the identification of replication initiation sites genome-wide (Leonard and Mechali 2013). Comparison of the replication initiation sites in S2, BG3, and Kc cells revealed that 16–20% of initiation sites are common to all three cell types, whereas 35–45% of activated origins are common to at least two cell types (Comoglio *et al.* 2015). These results highlight the cell-type specificity of origin sites, although an appreciable number of common origin sites exists as well.

Labeling of synchronized *Drosophila* cells *in vitro* with the nucleotide analog 5-bromo-2′-deoxyuridine (BrdU) coupled to microarray analysis revealed that distinct regions of the genome are replicated at different times during S phase. Most origins could be classified as early or late replicating origins with minimal overlap (MacAlpine *et al.* 2004; Eaton *et al.* 2011). Early replicating sites are correlated with increased chromatin accessibility (Bell *et al.* 2010; MacAlpine *et al.* 2010; Comoglio *et al.* 2015). In a survey of Kc, S2, and BG3 cells, it was found that replication timing profiles, or the temporal program in which regions of the genome are replicated in S phase, are largely correlated between these cell types, suggesting that replication timing is relatively conserved across different cell types (Lubelsky *et al.* 2014). Early replicating sequences are associated with activating chromatin marks such as H4K16ac, H3K79me1/2, H3K4me1/2/3, H3K27ac, and H3K18ac, ORC binding (see below), high gene density, and high gene expression. In contrast, late replicating sequences are associated with repressive chromatin marks such as H3K27me3 and H3K9me2/3 (Lubelsky *et al.* 2014). Furthermore, origins themselves are generally enriched for several histone modifications, including H3K9me1, H3K23me1, and H4K20me1 (Comoglio *et al.* 2015). Finally, origins are generally found to be enriched in GC content, suggesting that DNA shape and structure may play an important role in origin specification (Cayrou *et al.* 2011; Comoglio *et al.* 2015).

ORC binding has served as a useful marker for potential origins, as its localization to chromatin is necessary to recruit the replication machinery to initiate replication. In S2 cells, tethering ORC to various chromosomal sites is sufficient to direct replication initiation (Crevel and Cotterill 2012). In budding yeast, ORC binding is directed to the autonomously replicating sequence (ARS), a consensus sequence that is found at all origins of replication (Bell and Stillman 1992; Costa *et al.* 2013). In metazoans, ORC exhibits little to no sequence specificity both *in vitro* and *in vivo* (Vashee *et al.* 2003; Remus *et al.* 2004; MacAlpine *et al.* 2010; Miotto *et al.* 2016). Instead, ORC binds preferentially to negatively supercoiled DNA templates *in vitro*, providing evidence that DNA topology rather than DNA sequence governs ORC binding (Remus *et al.* 2004). ORC2 mapping in asynchronous *Drosophila* Kc167 cells revealed that ORC density is significantly higher at sites that initiate replication early in S phase, suggesting that replication timing is established in part at the level of ORC binding (MacAlpine *et al.* 2004, 2010). Additionally, ORC is significantly enriched at active promoters, raising the possbility that the local chromatin environment established at actively transcribed genes allows for ORC recruitment. ORC binding at transcription start sites is correlated with an enrichment for H3K9ac, H3K27ac, H3K4me2, and H3K4me3, histone modifications commonly found at active promoters. Likewise, these ORC binding sites are anticorrelated with the presence of the heterochromatic histone marks H3K9me2/3 and H3K27me3 (Eaton *et al.* 2011). Furthermore, ORC binding sites are enriched in the histone variants H3.3 and H2Av. They are depleted of bulk nucleosomes, both at sites of active transcription as well as sites not associated with an active promoter, emphasizing the idea that ORC localization is largely dictated by an open and dynamic chromatin environment (MacAlpine *et al.* 2010). Consistent with this idea, ORC binding sites are also highly enriched for ISWI, a member of the NURF chromatin remodeling complex (Eaton *et al.* 2011).

ORC binding appears to be regulated in part by chromatin remodeling. In pupae and S2 cells, binding sites of the insulator protein Suppressor of Hairy wing, or Su(Hw), are associated with the localization of members of the SAGA histone acetyltransferase complex as well as with OSA, a member of the Brahma (SWI/SNF) chromatin remodeling complex (Mazina *et al.* 2013; Vorobyeva *et al.* 2013). In *su*(*Hw*) mutants, enrichment of these factors is decreased at these insulator binding sites, concomitant with a higher enrichment of histone H3. Interestingly, ORC3 enrichment at these sites also is decreased in the *su*(*Hw*) mutant (Mazina *et al.* 2013), posing the possibility that Su(Hw) may recruit these chromatin remodeling factors to create a platform for ORC binding. Similar associations are observed with the CTCF, GAF, and BEAF32 chromatin insulator proteins, thus general chromatin remodeling may be associated with ORC binding (Vorobyeva *et al.* 2013). Intriguingly, Su(Hw) coimmunoprecipitates with ORC3, and artificial tethering of Su(Hw) to an ectopic site is sufficient for the recruitment of chromatin remodeling factors as well as ORC (Vorobyeva *et al.* 2013), providing further support for the establishment of an open chromatin environment in specifying ORC binding in *Drosophila*.

Methylation of H4K20 has been suggested to play important roles in replication initiation in mammalian cells by promoting the localization of ORC to replication origins (Jorgensen *et al.* 2007; Tardat *et al.* 2007, 2010; Houston *et al.* 2008; Beck *et al.* 2012; Kuo *et al.* 2012). In *Drosophila*, decreased activity of PR-Set7, the methyltransferase responsible for H4K20 monomethylation, results in DNA damage checkpoint activation and a lengthened S phase in neuroblasts (Sakaguchi and Steward 2007) and S2 cells (Sakaguchi *et al.* 2012). Consistent with these findings, Kc cells inhibited for H4K20 methylation exhibit a perturbed cell cycle with gross DNA damage, suggesting a defect in DNA replication (Li *et al.* 2016). Surprisingly, the inhibition of H4K20 methylation does not alter the genome-wide pattern of replication origin activation, but rather sensitizes late replicating domains to DNA damage. These results provide evidence that the primary role of H4K20 methylation in *Drosophila* is not to direct the recruitment of ORC to replication origins, but rather to ensure the integrity of late replicating domains during S phase.

### Developmental regulation of DNA replication in Drosophila

It also has become increasingly clear that in metazoans, the replication and developmental programs are tightly linked (Nordman and Orr-Weaver 2012). In addition to the possibility of merging genetic and biochemical techniques, developmental events themselves in *Drosophila* provide experimental advantages. This is because the properties of S phase and origin usage change as extensive cell cycle changes are employed during *Drosophila* development. In addition, inhibition of replication or increased replication at specific genomic sites in response to developmental cues provides models to decipher the regulation of replication origins and replication fork progression. First we address the developmental changes in S phase and origin localization. In the following section, copy number changes that provide models for replication origins and forks are discussed.

#### Developmentally regulated S phase changes:

*Drosophila* development is tightly linked to changes in the cell cycle and DNA replication programs. Rapid early embryogenesis, in the first 2 hr after fertilization, is achieved by accelerated DNA replication. In early embryos, nuclei divide quickly with no defined gap phases, an S phase length of ∼4 min, and replication origins spaced <10 kb apart (Blumenthal *et al.* 1974). This is in stark contrast, for example, to the larval brain and imaginal disc cells that can exhibit S phases lasting many hours, with origins of replication spaced >100 kb apart (Spradling and Orr-Weaver 1987). The high density of replication origins in early embryos likely reflects differences in chromatin structure and possibly the parameters of ORC binding, but this remains to be explored.

S-phase length gradually but moderately increases through the first 13 cell cycle divisions, and after the 13th division cycle a G2 gap phase is introduced, and S phase is dramatically lengthened to 40–50 min. This is correlated with changes in chromatin structure in which heterochromatin is formed (Shermoen *et al.* 2010), but how this impacts ORC binding, origin activation, and fork progression has yet to be determined. Notably, in embryonic division cycles 14–16, although a G2 phase is present, there is no detectable G1 phase (Foe and Alberts 1983; Foe 1989; Edgar and O’Farrell 1990; Knoblich *et al.* 1994). Thus resetting of origins must occur in G2 when Cyclin/CDK levels are high, or else abruptly as the chromosomes decondense in telophase.

**Figure 2:**
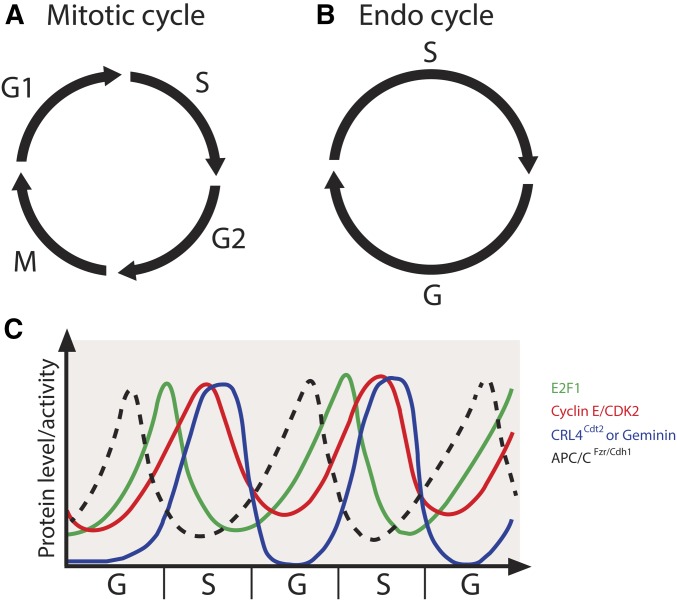
The oscillatory levels of key factors in endocycle maintenance. (A) The canonical mitotic cell cycle is composed of four sequential phases: G1, S, G2, and M. This cell cycle gives rise to two identical daughter cells. (B) The endocycle is composed of two alternating phases: G and S. This leads to increased DNA ploidy within a single cell. (C) The endocycle is driven by oscillations in key cell cycle factors. Levels of the E2F1 transcription factor rise at the end of G phase to turn on transcription of *cycE* and is degraded during S phase. Cyclin E/CDK2 activity rises at the start of S phase to initiate DNA replication and falls at the end of S phase after the completion of replication. The activity of the E3 ubiquitin ligase CRL4-Cdt2 peaks in S phase, when it marks E2F1 for degradation. In addition, the activity of APC/C^Fzr/Cdh1^ peaks when Cyclin E/CDK2 activity levels are low and targets Geminin for degradation during G phase.

Most cells in the embryo cease mitotic divisions after the 16th division cycle and enter a variant cell cycle called the endocycle (also referred to as endoreduplication) that continues through larval and adult development (Painter and Reindorp 1939; King and Burnett 1959; Balls and Billett 1973; Hammond and Laird 1985a,b; Smith and Orr-Weaver 1991; Lilly and Duronio 2005). The neural and imaginal tissues are the only tissues that continue to divide mitotically during embryonic and larval development. The endocycle consists of alternating S and G phases (Figure 2B) without mitosis and cell division that occur during the canonical cell cycle (Figure 2A). During the endocycle, DNA content is increased at the genomic level, thus producing polyploid cells. As organism size is greatly increased throughout larval development, polyploidy is thought to coordinate cell size and tissue growth by generating large, highly metabolically active cells (Edgar *et al.* 2014; Orr-Weaver 2015). Indeed, blocking polyploidization inhibits cell and larval growth, inhibiting normal tissue function (Edgar and Orr-Weaver 2001).

The endocycle is utilized throughout the plant and animal kingdoms, indicating the importance of this variant cell cycle during development across organisms (Orr-Weaver 2015). Key insights into the regulation of the endocycle and its coordination with the replication program have derived from seminal studies in *Drosophila*. Nearly all larval tissues and many adult tissues in *Drosophila* have increased ploidy that is achieved via the endocycle. The replicated DNA duplex copies are held in register to produce polytene chromosomes with stereotypic banding patterns in most *Drosophila* endocycling tissues. The most well studied of these polyploid tissues is the larval salivary gland, which undergoes ∼10 endocycles during larval development to obtain a final ploidy of roughly 1024C (Hammond and Laird 1985b). During the endocycle, cells must suppress the mitotic machinery to prevent entry into the mitotic program and subsequent cell division. One strategy that endocycling cells use to achieve this is to downregulate the activity of mitotic Cyclins and mitotic CDKs at the transcriptional level. At the switch from the mitotic cell cycle to the endocycle, cells in the embryo cease expression of the mitotic regulators Cyclin A, Cyclin B, Cyclin B3, String/Cdc25, and CDK1 (Sauer *et al.* 1995; Maqbool *et al.* 2010). However, the developmental signals that regulate transcription of these regulators at this switch are not well understood.

In *Drosophila*, Cyclin E/CDK2 activity is the major driver of S-phase entry. Mutations in the *cycE* gene inhibit DNA replication in both mitotic and endocycling cells (Knoblich *et al.* 1994). Importantly, continuous overexpression of cyclin E in the salivary gland blocks endocycling, suggesting that oscillations in Cyclin E/CDK2 activity are required for continued endocycling (Follette *et al.* 1998; Weiss *et al.* 1998). The oscillatory expression of *cycE* is mediated by oscillations in the levels of the transcription factor E2F1, which reaches high levels during G phase and is degraded at the end of S phase (Zielke *et al.* 2011). E2F1 degradation is mediated by the E3 ubiquitin ligase CRL4-Cdt2 (Shibutani *et al.* 2007, 2008), whose activity peaks during S phase (Zielke *et al.* 2011) (Figure 2C). Artificial stabilization of E2F1 prevents endocycling in the salivary gland, indicating that E2F1 degradation is required for continued endocycling (Zielke *et al.* 2011). At the end of S phase, degradation of E2F1 is followed by ubiquitin-dependent degradation of Cyclin E via the E3 ubiquitin ligase CRL1-Ago along with its activator Minus (Shcherbata *et al.* 2004; Szuplewski *et al.* 2009; Zielke *et al.* 2011). The degradation of Cyclin E allows for the completion of S phase and the relicensing of replication origins in the subsequent G phase. Additionally, oscillations of the *Drosophila* CDK2 inhibitor Dacapo peak similarly to E2F1 during G phase of the endocycle (Hong *et al.* 2003, 2007). Dacapo contributes to the attenuation of Cyclin E/CDK2 activity during G phase and is subsequently degraded during S phase via its PIP degron (Swanson *et al.* 2015). Although Dacapo is not necessary for the endocycle (Hong *et al.* 2003; Zielke *et al.* 2011), its overexpression inhibits the endocycle, suggesting that Dacapo plays a role in establishing the Cyclin E/CDK2 activity threshold necessary to trigger S phase (Shcherbata *et al.* 2004; Hong *et al.* 2007; Zielke *et al.* 2011; Swanson *et al.* 2015).

Much like during the archetypal cell cycle, endocycling cells must also prevent rereplication during S phase. In the mitotic cell cycle, helicase loading at origins is restricted to late M through G1 phase. At the G1/S transition, the activities of S phase CDK and DDK increase dramatically, allowing for the assembly and activation of the replicative helicase complex to begin DNA replication (Costa *et al.* 2013). After S-phase onset, high S-phase CDK activity prevents the reloading of the helicase complex at origins that have already fired by inhibiting the activity of several replication initiation proteins required to load the helicase onto origin DNA (Blow and Dutta 2005). For example, phosphorylation of the DUP/Cdt1 replication initiation factor by Cyclin E/CDK2 during S phase promotes DUP/Cdt1 degradation in mitotic and endocycling cells (Thomer *et al.* 2004). DUP/Cdt1 protein levels oscillate during the endocycle (Hong *et al.* 2007), and DUP/Cdt1 protein was found to accumulate in the G phase and rapidly decrease once cells enter into S phase (Whittaker *et al.* 2000; Thomer *et al.* 2004). Finally, constitutive overexpression of DUP/Cdt1 is sufficient to induce polyploidy in wing disc cells and results in enlarged nuclei with increased DNA content in endocycling follicle cells, emphasizing the significance of the regulation of DUP/Cdt1 levels by Cyclin E/CDK2 in preventing rereplication (Thomer *et al.* 2004).

In *Drosophila* as well as in other metazoans, Geminin is an inhibitor of helicase loading and exhibits high levels during the S phase in the archetypal cell cycle to prevent rereplication (Quinn *et al.* 2001). During M phase, Geminin is targeted for degradation by the anaphase promoting complex (APC)/cyclosome, allowing for helicase loading in the subsequent G phase (McGarry and Kirschner 1998). In a similar manner, Cyclin E/CDK2 activity peaks during S phase in the endocycle (Figure 2C). Additionally, Geminin levels oscillate during the endocycle, with low levels in G phase to allow for helicase loading and high levels in S phase to prevent reloading of helicases and rereplication. Geminin is targeted for degradation at the end of the endocycle S phase by the APC/cyclosome through the APC activator Fzr/Cdh1, and APC/C^Fzr/Cdh1^ activity is inhibited by Cyclin E/CDK2 activity (Narbonne-Reveau *et al.* 2008; Zielke *et al.* 2008) (Figure 2C). The oscillation of the activity level of Geminin is required for the endocycle, as constitutive expression of Geminin inhibits endocycle progression (Zielke *et al.* 2008). However, Geminin is not essential for salivary gland development (Zielke *et al.* 2011), suggesting that multiple overlapping mechanisms exist to prevent rereplication in endocycling cells.

#### Tissue specificity of Drosophila origins:

To date, the polytene larval salivary gland is the only differentiated tissue undergoing genomic replication in which genome-wide ORC localization has been reported (Sher *et al.* 2012). In a survey of ORC binding in Kc, S2, and Bg3 cells, it was found that about a third of the identified ORC binding sites were shared between all three cell types (Eaton *et al.* 2011). Similarly, 31% of the ORC binding sites identified in the larval salivary gland are common with all three cell lines, indicating that a significant level of ORC binding site conservation may exist not only in cell culture lines but in differentiated tissues as well. Notably, 28% of the salivary gland ORC binding sites are unique to this tissue. Consistent with cell culture studies, 73% of the salivary gland ORC binding sites are within a kilobase of a transcription start site. A total of 57% of the salivary gland-specific ORC binding sites are found near a transcription start site, but the genes controlled by these promoters are not uniquely expressed in the salivary gland. Thus, tissue-specific expression of genes does not correlate with tissue-specific ORC binding (Sher *et al.* 2012).

## Insights into Regulation of DNA Replication from Localized Changes in DNA Copy Number

Interestingly, increases in gene copy number in polyploid *Drosophila* cells are not uniform throughout the genome. Heterochromatin is repressed for replication in many *Drosophila* polyploid cells, and in several larval tissues, defined eukaryotic genomic regions have been shown to be underreplicated (UR) relative to overall ploidy of the cell (Hammond and Laird 1985a,b; Nordman *et al.* 2011). Additionally in the adult female, follicle cells complete endocycling and begin gene amplification, leading to specific sites within the genome that are increased in copy number (Spradling 1981). The study of underreplication and differential gene amplification in *Drosophila* has provided important understanding about the developmental regulation of both origin activation and fork progression at the molecular level. In the following section, we summarize our current understanding of the molecular parameters of DNA replication from analysis of differential DNA replication.

### Underreplication and local copy number reduction

**Figure 3:**
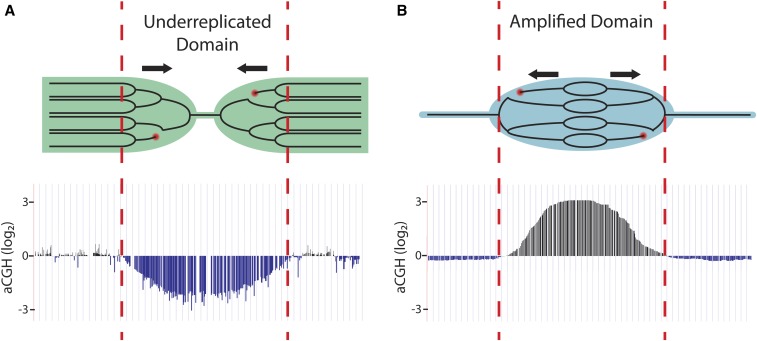
Differential DNA replication. (A) Underreplication results from two effects: absence of replication origins and initiation within a domain coupled with impaired progression of replication forks initiating from flanking origins (arrows indicate direction of fork progression). Replication forks are destabilized within underreplicated regions and can lead to double-stranded DNA breaks (red circles). These can lead to deletions or rearrangements in the region. Array-based comparative genome hybridization (aCGH) analysis of an underreplicated site indicates decreased copy number relative to overall ploidy. aCGH data are modified from Sher *et al.* (2012). (B) In endocycling follicle cells, developmentally programmed gene amplification occurs through repeated replication origin firing followed by bidirectional fork progression away from the origin (arrows indicate direction of fork progression). Rereplication can lead to fork collisions, resulting in double-strand DNA breaks (red circles). aCGH analysis of an amplified site indicates a gradient of increased copy number relative to overall ploidy spanning 100 kb, with the highest copy number at the origin of replication. aCGH data are modified from Kim *et al.* (2011).

Although polytene cells have increased DNA content per cell, gene copy number is not uniform throughout the genome. For instance, it has long been known that the heterochromatic regions in polyploid salivary gland, follicle cell, and nurse cell chromatin are reduced in copy number relative to overall ploidy, a phenomenon known as underreplication (Zhimulev *et al.* 1982; Hammond and Laird 1985a,b; Lamb and Laird 1987; Smith and Orr-Weaver 1991) (Figure 3A). In addition to heterochromatin, array-based comparative genome hybridization (aCGH) and high-throughput genomic sequencing studies have revealed that larval salivary gland, midgut, and fat body tissues contain precise euchromatic regions that are underreplicated as well (Belyakin *et al.* 2005; Nordman *et al.* 2011; Sher *et al.* 2012; Yarosh and Spradling 2014). These euchromatic UR regions can be large, ranging up to 450 kb in size. They exhibit features of repressed chromatin and thus also are termed intercalary heterochromatin, although as noted below these regions are not necessarily repressed for transcription (Belyaeva *et al.* 2008; Filion *et al.* 2010). Only a third of identified UR regions are common to all the three tissues, highlighting the high degree of tissue specificity of underreplication (Nordman *et al.* 2011).

In addition to genome-wide profiling approaches in *Drosophila* cell culture, the study of underreplication in *Drosophila* polyploid tissues has uncovered important links between differentiation, development, and the control of DNA replication. Notably, ORC is bound throughout most of the salivary gland genome but is excluded within UR regions (Sher *et al.* 2012). This finding strongly suggests that replication initiation does not occur within these regions, and thus replication of these regions is dependent upon replication forks emanating from outside the region. Interestingly, these UR regions are devoid of RNA polymerase II, strongly inhibited for transcription, and are enriched for the heterochromatic chromatin mark H3K27me3 (Sher *et al.* 2012). These results are consistent with the idea that UR regions in the salivary gland represent repressive chromatin domains that are inhibitory to both transcription and DNA replication initiation. Indeed, nearly all of the UR regions in the salivary gland correspond to domains of repressive chromatin as defined in genome-wide chromatin landscape studies (Filion *et al.* 2010; Kharchenko *et al.* 2011; Yarosh and Spradling 2014). UR regions in the larval fat body also are devoid of ORC binding, suggesting that ORC repression in these domains may be a common feature of underreplication (B. Hua, H. Kashevsky, G. Bell, J. Von Stetina, and T. Orr-Weaver, unpublished data). The analysis of UR regions in fat body shows, however, that underreplication is not causally linked to a chromatin state that is repressive for transcription, because the genes present in URs in the fat body are robustly transcribed (Nordman *et al.* 2011).

Interestingly, *orc1* and *orc2* null mutant salivary glands continue the endocycle, though they reach ploidy levels two- to fourfold lower than wild-type salivary glands (Park and Asano 2008; Sher *et al.* 2012). These results indicate that the endocycle can occur to a significant extent in the absence of newly synthesized ORC1 and ORC2. However, *orc1* and *orc2* mutants exhibit a marked change in the underreplication pattern in the salivary gland where all but the most pronounced UR regions become fully replicated (Sher *et al.* 2012). Thus, ORC plays an important role in the distribution of replication along polyploid chromosomes, and it is possible that replication in the *orc1* and *orc2* mutants is allowed by maternal loading of ORC or by residual activity of ORC missing the ORC1 or ORC2 subunits.

Underreplication has been most extensively studied in *Drosophila*, but underreplication of defined euchromatic regions occurs outside of Diperta as well. A total of 47 regions of the genome in the polyploid mouse trophoblasts giant cells are recurrently and reproducibly underreplicated, although fold underreplication levels are low compared to that observed in *Drosophila*, with most of the identified regions being less than twofold reduced in copy number and thus not called by the cut-off criteria used in *Drosophila* (Sher *et al.* 2013; Hannibal *et al.* 2014). Nevertheless, this highlights the importance and relevance of studying underreplicated regions in fly polyploid tissues as a model for differential replication in polyploid tissues outside of *Drosophila*.

#### Inhibition of fork progression by SUUR:

**Figure 4:**
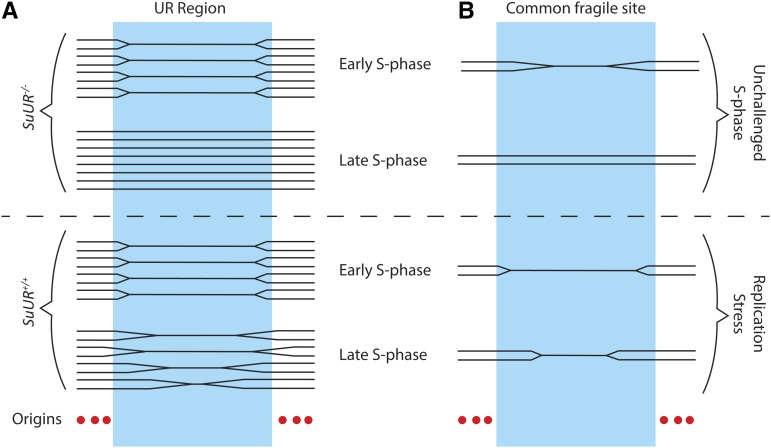
Underreplication as a model for human common chromosomal fragile sites. (A) In polyploid *Drosophila* larval tissues, underreplicated (UR) domains are largely devoid of replication origins and initiation events and depend on forks emanating from outside of the domain for their replication. Underreplication is dependent upon the SUUR protein, and loss of SUUR activity results in full replication of all UR regions. (B) The human common fragile site FRA3B also is devoid of replication initiation events in some tissues and depends on forks emanating from outside the 700-kb region for its replication. Under replication stress, forks fail to fully replicate the domain, leading to unreplicated DNA and chromosome fragility.

Underreplication in the salivary gland, fat body, and midgut are dependent upon the **Su**ppressor of **U**nder**r**eplication protein (SUUR), as all underreplicated regions become fully replicated in the *SuUR* mutant (Nordman *et al.* 2011; Sher *et al.* 2012). SUUR is a chromatin protein that as of yet has not been identified outside of *Drosophila* (Nordman and Orr-Weaver 2015). Loss of SUUR function does not restore ORC binding in the underreplicated regions of the salivary gland, indicating that SUUR does not act at the level of replication initiation to inhibit replication (Sher *et al.* 2012). Instead, *SuUR* mutants exhibit enhanced rates of replication fork progression, suggesting that SUUR acts to inhibit replication fork progression (Sher *et al.* 2012). These findings support a model in which underreplicated domains are dependent upon replication forks emanating from origins outside of the region, and underreplication is achieved by the SUUR-mediated inhibition of fork progression through these domains (Figure 4A).

Subsequent studies revealed that SUUR coimmunoprecipitates with the sliding clamp PCNA and the replication fork factor CDC45 in embryonic nuclear extracts and tracks with the replication fork in follicle cells undergoing gene amplification (detailed in subsequent sections), further supporting the fact that SUUR is recruited to active replication forks (Kolesnikova *et al.* 2013; Nordman *et al.* 2014). Consistent with studies in endocycling tissues, *SuUR* mutants exhibit significantly enhanced fork progression in amplifying follicle cells, and overexpression of SUUR severely hampers fork progression (Nordman *et al.* 2014). Together, these results indicate that SUUR is a general inhibitor of fork progression and acts directly at the replication fork. However, the molecular mechanism of fork inhibition by SUUR remains to be elucidated.

Two key questions are whether SUUR inhibits replication in the pericentric heterochromatin and in the dispersed UR regions by the same mechanism, and how SUUR becomes recruited to replication forks in the UR regions. Recent findings on the dynamics of histone H1 on salivary gland chromosomes during the endocycle provide insights (Andreyeva *et al.* 2017). This histone is necessary for underreplication both in the pericentric heterochromatin and the UR regions. H1 is required for SUUR localization on chromosomes, and the two proteins directly bind each other. Interestingly, early in S phase in the endocycle, H1 is enriched at regions that will replicate late, including those that become underreplicated. Later in the endocycle S phase, H1 becomes more uniformly distributed on the chromosomes. These results provide one mechanism for the regional specificity of SUUR action: that it is directed to specific regions by the presence of H1 histone. This is not sufficient, however, as SUUR localization and underreplication occur at only a subset of H1 localization sites on the euchromatic arms.

#### Fork instability and DNA damage in UR regions:

In the polytene chromosomes of the salivary gland, UR domains are cytologically enriched for a key marker of double-stranded DNA breaks (DSBs), γH2Av (Andreyeva *et al.* 2008). Chromatin immunoprecipitation studies revealed that γH2Av is enriched throughout the entire region of each UR domain, indicating that UR domains are prone to DNA damage (Nordman *et al.* 2014). Enrichment of γH2Av in these UR regions is dependent upon SUUR function, suggesting that DNA damage in these regions is caused by fork instability mediated by SUUR. Additionally, high-throughput sequencing and analysis of read pairs generated from salivary gland DNA indicate that large deletions ranging 10–500 kb in size may result from DNA damage and local repair in these regions (Yarosh and Spradling 2014).

#### Potential biological functions of underreplication:

As *SuUR* mutants are viable and exhibit normal morphology and fertility (Belyaeva *et al.* 1998), it remains unclear to what extent SUUR is required in normal development. Given that SUUR is a general inhibitor of fork progression, it is possible that SUUR serves to provide an extra level of regulation to ensure proper replication timing in the genome. SUUR may regulate replication timing during S phase by blocking fork progression to ensure that regions of the genome are not replicated until late in S phase. Another function of SUUR could be to distribute termination events throughout the genome (Hawkins *et al.* 2013). Although these would seem to be critical roles, they may not be essential unless the cells become subject to replication stress.

Because UR regions become fully replicated in *SuUR* mutants, the biological role of underreplication remains to be elucidated. The UR regions in the salivary gland are enriched in genes involved in cell adhesion, segmentation, transcription factor activity, programmed cell death, mesoderm development, and cell motility (Sher *et al.* 2012; Yarosh and Spradling 2014). Additional regions that are consistently underreplicated but to lower extents in the salivary gland are highly enriched in immunoglobulin superfamily genes and genes involved in the nervous system (Yarosh and Spradling 2014). Strikingly, transcription of genes within UR regions is largely repressed in the larval salivary gland and midgut tissues, suggesting that decreased copy number may cause lower gene expression (Nordman *et al.* 2011; Sher *et al.* 2012). However, in the *SuUR* mutant in which UR regions are fully replicated, gene expression within the UR regions remains repressed, demonstrating that underreplication is not required for transcriptional repression in these domains (Nordman *et al.* 2011; Sher *et al.* 2012). Additionally, many genes within the UR regions in the fat body are significantly transcribed, thus underreplication and the repression of transcription can be mechanistically uncoupled (Nordman *et al.* 2011).

As deletions and rearrangements have been reported throughout UR regions, one proposed role for underreplication is to promote the somatic diversity of genes within these domains (Yarosh and Spradling 2014). This idea is especially interesting in the context of the immunoglobulin superfamily genes found in some UR sites in which gene rearrangements may be advantageous. Nevertheless, the biological role of underreplication has yet to be fully uncovered.

#### Underreplication as a model for common chromosomal fragile sites:

In addition to its utility in understanding the mechanisms underlying differential replication inhibition, underreplication in *Drosophila* polyploid tissues serves as a promising model system for human common chromosomal fragile sites. Common fragile sites (CFSs) are chromosomal locations characterized by recurrent breaks, gaps, and constrictions on metaphase chromosomes upon replication stress (Durkin and Glover 2007). CFSs often are found in euchromatin and extend over megabase-long regions of the chromosome (Schwartz *et al.* 2006; Smith *et al.* 2006). It appears that multiple mechanisms can lead to CFSs, but one of these involves replication origins and fork progression (Ozeri-Galai *et al.* 2014). For example, replication initiation does not occur within a 700-kb region forming the core of the most active human CFS, FRA3B (Letessier *et al.* 2011). Thus, replication of this large region is dependent entirely upon replication forks emanating from origins of replication outside of this domain. A general challenge to replication forks results in incomplete replication of the FRA3B domain, leading to chromosome fragility and instability (Figure 4B). UR regions in the *Drosophila* salivary gland are also devoid of origins of replication and rely on forks coming from flanking regions for their replication (Figure 4A). Additionally, UR regions are prone to DNA damage, a property common to CFSs. Combining the genetic and cell biological toolkits of the *Drosophila* system with genome-wide profiling techniques will allow for deeper understanding of the mechanisms that underlie replication initiation repression in these regions, control of fork progression, and the molecular properties of CFSs in human cells.

### Developmentally programmed follicle cell gene amplification to increase local copy number

While the underreplication system has allowed study of replication properties and dynamics across large, defined chromatin domains, the molecular dissection of the mechanisms that underlie origin activation requires the study of well-defined origins of replication. Additionally, it is necessary to know when single origins fire in order to study individual origin activation events. The study of *Drosophila* follicle cell gene amplification has allowed the isolation and detailed molecular characterization of single metazoan origins of replication. In this section, we review the characteristics of follicle cell gene amplification and focus on key studies that have led to critical understanding of the molecular parameters that regulate origin activation and fork progression.

To date, aCGH analyses have been performed on seven distinct *Drosophila* tissues to assay differential DNA replication genome-wide (Kim *et al.* 2011; Nordman *et al.* 2011; Sher *et al.* 2012; B. Hua, H. Kashevsky, G. Bell, J. Von Stetina, and T. Orr-Weaver, unpublished results). Of the examined tissues, only the ovarian somatic follicle cells have been found to exhibit gene amplification or increased copy number of distinct genomic regions relative to overall ploidy of the cell.

In the adult female fly, the somatic follicle cells form an epithelial cell layer around the developing oocyte in the egg chamber and are responsible for the production of egg shell proteins that are important for the integrity of the chorion (Spradling 1993). Oogenesis proceeds in egg chambers composed of polyploid nurse cells, the oocyte, and surrounding follicle cells. Egg chamber stages can be distinguished morphologically as development progresses (Spradling 1993). The follicle cells, derived from the follicle cell stem cell population, undergo mitotic divisions until stage 6 of development, resulting in ∼1000 follicle cells on a single egg chamber. Follicle cells begin the endocycle at stage 7, performing three asynchronous rounds until the end of stage 9. By stage 10A, all follicle cells have completed endocycling and nearly all have 16C genome content. At stage 10B, genome-wide replication shuts off, and specific origins in each follicle cell synchronously begin gene amplification (Calvi *et al.* 1998). During gene amplification, amplicon origins undergo repeated firing through a rereplication-based mechanism, generating a series of bidirectional replication forks that progress 50 kb to both sides of the origin (Spradling 1981; Claycomb *et al.* 2002). This results in a gradient of amplified DNA, with the highest copy number at the origin of replication (Figure 3B). Gene amplification continues until stage 13, and follicle cells are ultimately sloughed off the egg chamber at the end of oogenesis.

Most amplicon loci contain genes encoding critical protein components of the egg shell or proteins involved in the integrity of the chorion (Spradling 1981; Claycomb *et al.* 2004; Fakhouri *et al.* 2006; Kim and Orr-Weaver 2011; Kim *et al.* 2011; Tootle *et al.* 2011) (Table 2). Gene amplification is used as a developmental strategy to increase the template copy number for key chorion components whose protein products must be produced quickly in a relatively short developmental time window (∼7.5 hr). Female-sterile alleles of essential replication factors demonstrate the requirement of ORC2 (Landis *et al.* 1997), MCM6 (Schwed *et al.* 2002), DUP/Cdt1 (Whittaker *et al.* 2000), Chiffon/Dbf4 (Landis and Tower 1999), and MUS101/TopBP1 (Komitopoulou *et al.* 1983; Orr *et al.* 1984; Yamamoto *et al.* 2000) during gene amplification and egg development, indicating that gene amplification in the follicle cells likely uses the same components as those during normal S phase. Additionally, as egg shell integrity is dependent upon proper execution of the follicle cell gene amplification program, the identification of thin egg shell mutants has been an important and powerful method to uncover key players in gene amplification using forward genetic approaches. These have included the conserved replication proteins noted above as well as new replication proteins such as Humpty Dumpty and the Claspin checkpoint protein (Landis *et al.* 1997; Landis and Tower 1999; Whittaker *et al.* 2000; Schwed *et al.* 2002; Bandura *et al.* 2005; Choi *et al.* 2017).

**Table 2:** *Drosophila* amplicons in follicle cells

Cytological location	Max fold amplification	Stages of origin firing	Genes involved in egg shell function
*7F*	18–20	10B–11	*Cp7Fa*, *Cp7Fb*, *Cp7Fc*, *Cp36*, *Cp38*
*22B*	4	10B–13	None
*30B*	4	10B	*CG11381*, *CG13113*, *CG13114**^a^*
*34B*	6	10B, 13	*Vm34Ca*
*62D*	4	10B, 13	*yellow-g*, *yellow-g2*
*66D*	60–80	10B–11	*Cp18*, *Cp15*, *Cp19*, *Cp16*

a Predicted chorion genes (Fakhouri *et al.* 2006; Tootle *et al.* 2011).

The gene amplification system has allowed the molecular characterization of single origins of replication, proving a powerful tool to dissect the mechanisms that underlie origin activation. During gene amplification, origin firing is tightly coordinated with follicle cell differentiation. Amplification is achieved by repeated rounds of origin firing that occur at defined developmental time points during follicle cell differentiation, permitting temporal and quantitative resolution of replication initiation events (Table 2). Furthermore, defined sets of replication forks are generated from these single origins of replication, allowing both the cytological and molecular characterization of replication fork progression in these cells (Claycomb *et al.* 2002). In the next sections, we summarize the key findings regarding the molecular mechanisms underlying origin activation and fork progression that have emerged from studying the gene amplification system.

#### Control of origin activation during gene amplification:

Through aCGH analysis of 16C follicle cells, six distinct sites of amplification have been identified (Kim *et al.* 2011). These sites, termed *Drosophila* amplicons in follicle cells (*DAFC*s), are located at distinct sites within the follicle cell genome and are referred to by their cytological locations. The level of gene amplification varies, ranging from 60- to 80-fold amplification at *DAFC-66D* to 4-fold amplification at several amplicons (Spradling 1981; Claycomb *et al.* 2004; Kim *et al.* 2011) (Table 2).

Genome-wide ORC mapping from amplification-stage egg chambers revealed that ORC is enriched at all six amplification origins in broad domains ranging from 12 to 32 kb in size (Kim *et al.* 2011). Significant ORC binding was detected at nonamplified regions as well, revealing that ORC binding alone is not sufficient for origin activation during gene amplification. Further analysis on genome-wide ORC binding from purified amplifying follicle cells will be necessary, however, to rule out the possibility these sites of enrichment are derived from the nurse cells or the oocyte of the egg chamber. Interestingly, roughly two-thirds of the identified ORC binding sites overlapped with transcription units, consistent with ORC localization studies in cell culture. However, only a 10th of these ORC binding sites are associated with genes that are expressed at high levels [reads per kilobase per million (RPKM) >3], in contrast to cell culture studies in which most ORC binding sites overlap with active promoters (MacAlpine *et al.* 2010).

Many studies have profiled the underlying chromatin signature at amplicon origins. The use of both cytological and molecular techniques have revealed that amplicon origin activity is correlated with a significant enrichment of histone acetylation marks, namely AcH3, H4K5ac, H4K8ac, H4K12ac, and H4K16ac (Aggarwal and Calvi 2004; Hartl *et al.* 2007; Kim *et al.* 2011; Liu *et al.* 2012; McConnell *et al.* 2012). Tethering of the histone deacetylase Rpd3 to a transgenic amplicon origin significantly reduces its activity (Aggarwal and Calvi 2004; Kim *et al.* 2011), whereas tethering of the histone acetyl transferase HBO1 increases its activity (Aggarwal and Calvi 2004), indicating that histone acetylation plays an important local role in modulating origin activity. As histone acetylation also is correlated with transcriptional activity, it is thought that these histone modifications serve to establish an open chromatin environment that is conducive to the recruitment and loading of the large protein complexes involved in transcription as well as DNA replication.

In *Drosophila* S2 cells, the histone variants H3.3 and H2Av are enriched at ORC binding sites (MacAlpine *et al.* 2010). In follicle cells, H3.3 is abundant at the amplicon sites before and during amplification, overlapping with ORC binding regions (Paranjape and Calvi 2016). *H3.3* null mutant flies, however, carry out genomic replication and gene amplification without detectable defects. Thus H3.3 is not essential for origin activation in these cells. These results suggest that although H3.3 is not required for origin activation, it may serve as a marker, possibly along with other histone variants and modifications, for chromatin attributes important for origin function and replication initiation.

Recently, nucleosome density and position have been investigated as regulators of ORC binding and replication initiation at the gene amplification loci. In budding yeast, nucleosomes are strictly and reproducibly positioned around the ARS consensus sequence at origins across the genome (Eaton *et al.* 2011; Belsky *et al.* 2015). In follicle cells, ORC binding regions at the *DAFC-66D* origin correspond to nucleosome-depleted regions (Liu *et al.* 2015), and ORC binding sites are generally depleted of nucleosomes in S2 cells as well (MacAlpine *et al.* 2010). ORC binding sites occur preferentially at AT-rich DNA sequences in amplifying follicle cells, suggesting that ORC binding to DNA is not solely a passive effect of the absence of nucleosomes, but rather favors the DNA regions that are disfavored by nucleosomes (Liu *et al.* 2015). This idea is consistent with the finding that replication initiation factor binding sites also tend to be AT-rich in cultured cells, and thus this property may be conserved across different replication contexts in *Drosophila* development (Comoglio *et al.* 2015). Nucleosome positioning in the follicle cells does not correlate with changes in amplicon origin activity, and nucleosome positioning at *DAFC-66D* is remarkably similar to that in the equivalent region in nonamplifying S2 cells. Therefore nucleosome positioning does not fully govern the specificity of ORC binding and origin activity in *Drosophila* (Liu *et al.* 2015). Rather, nucleosome positioning may be a passive effect of origin specification to allow for the binding of downstream replication initiation factors.

Individual characterization of the follicle cell amplicons has revealed that the activation of metazoan origins is regulated by an extremely diverse set of mechanisms. First, it was found that the *DAFC-66D* origin, *oriβ*, requires a 440-bp enhancer element called amplification control element for the third chromosome chorion cluster (*ACE3*) for activity (Orr-Weaver and Spradling 1986; Carminati *et al.* 1992). *ACE3* directs ORC binding at *oriβ*, located 1.5 kb away, to promote origin firing (Austin *et al.* 1999; Chesnokov *et al.* 1999). Additionally, normal *DAFC-66D* amplification requires the functions of Myb, Rb, and E2F1. E2F1 and Myb are both localized to *ACE3*, and an E2F1-Rb-ORC complex can be identified in ovary extracts, suggesting a direct role of these factors in regulating ORC activity during *DAFC-66D* origin activation (Bosco *et al.* 2001; Beall *et al.* 2002, 2004). Second, it was found that solely *DAFC-62D* exhibits transcription-dependent origin firing. Interestingly, transcription is required at *DAFC-62D* in *trans*, though this *trans*-acting mechanism has yet to be elucidated (Xie and Orr-Weaver 2008; Hua *et al.* 2014). Third, *DAFC-34B* is unique in that it exhibits origin firing at two separate stages of development, and the final round of origin firing occurs in the absence of detectable ORC localization. This raises the possibilities of ORC-independent origin firing or that origin firing can occur with dramatically reduced ORC enrichment or activity (Kim and Orr-Weaver 2011). Finally, *DAFC-22B* exhibits strain-specific amplification. Strikingly, relocation of a 10-kb fragment from the *22B* locus from a *22B* nonamplifying strain to an ectopic site restores *DAFC-22B* origin activity, indicating that the *DAFC-22B* origin is repressed in *cis* by an inhibitory chromosomal element at the endogenous location (Kim *et al.* 2011). Together, these studies highlight the diversity of mechanisms by which the activation of gene amplification origins is regulated.

**Figure 5:**
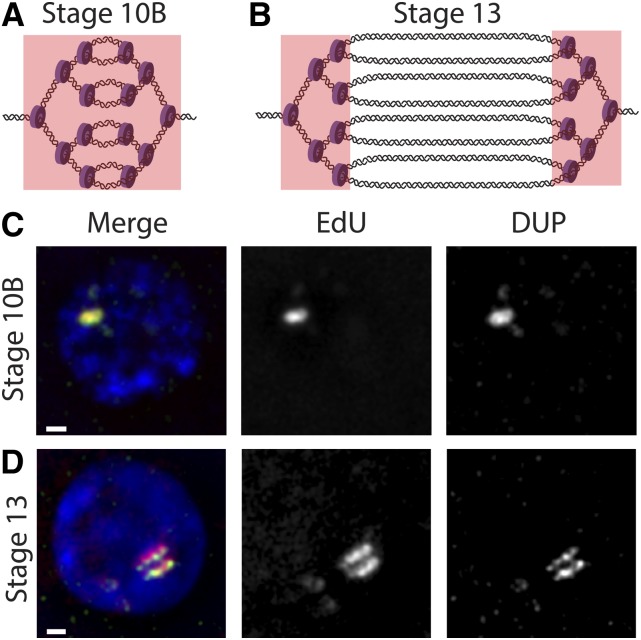
Visualization of replication forks during follicle cell gene amplification. (A) At stage 10B, genomic replication is shut off, and origin firing and fork elongation begin at the *DAFC-66D* amplicon (full copy number not depicted). EdU is incorporated throughout the amplicon (indicated by the pink box), resulting in a single focus of signal. (B) At stage 13, origin firing no longer occurs at *DAFC-66D* while replication forks continue to progress. EdU is incorporated at the two distinct sets of replication forks (pink boxes), resulting in a double bar of signal. (C) Immunofluorescence images of a stage 10B follicle cell nucleus depicting the localization of EdU (red), DUP (green), and DNA (blue). (D) Immunofluorescence images of a stage 13 follicle cell nucleus depicting the localization of EdU (red), DUP (green), and DNA (blue). Bar, 1 µm.

How is rereplication achieved during gene amplification? One possibility is that the replication initiation factor DUP fails to be inactivated and thus promotes reloading of the helicase and rereplication at the amplicons. During the archetypal S phase, DUP activity is restricted to late M and G1 phase through inhibition by the protein factor Geminin and by CRL4(Cdt2)-mediated degradation during S phase (Lee *et al.* 2010). At the most highly amplified locus, *DAFC-66D*, DUP is detectable cytologically in follicle cells well after the start of amplification and surprisingly tracks with replication forks (Claycomb *et al.* 2002) (Figure 5). Additionally, excessive DNA amplification is observed in the follicle cells in *geminin* mutants (Quinn *et al.* 2001), and stabilization of DUP protein leads to excessive DNA amplification and ectopic genomic replication (Thomer *et al.* 2004; Lin *et al.* 2009). One possibility for DUP persistence during gene amplification is that CRL4(Cdt2) ubiquitin ligase activity may be attenuated in the follicle cells during these developmental stages (Lee *et al.* 2010). Consistent with this idea, another target of the CRL4(Cdt2) ubiquitin ligase, E2F1, also persists through the start of amplification (Sun *et al.* 2008). Low CRL4(Cdt2) activity would allow for the continued presence of DUP even after the first round of origin activation at the amplicons, and this pool of DUP could permit helicase reloading and origin refiring.

#### *Drosophila* gene amplification as a tool to study fork progression:

In addition to its power in dissecting origin activation, the gene amplification system has allowed the study of the replication forks emanating from a single origin of replication both at the molecular and cellular levels. The bidirectional sets of replication forks originating from a single origin of replication can be tracked cytologically by labeling follicle cells with a thymidine analog such as BrdU or 5-ethynyl-2′-deoxyuridine (EdU) (Calvi *et al.* 1998; Claycomb *et al.* 2002). Sites of amplification can be specifically visualized by BrdU or EdU incorporation because genomic replication is shut off during gene amplification (Calvi *et al.* 1998; Claycomb *et al.* 2002). During the initial stages of amplification at *DAFC-66D*, replication initiation and fork elongation are coupled, which gives rise to a single focus of BrdU/EdU staining. However, during later stages of gene amplification, origin firing at *DAFC-66D* ceases, and BrdU/EdU foci solely mark nucleotide incorporation at the active replication forks on either side of the origin, resulting in double bars of BrdU/EdU signal (Figure 5). The MCM2–7 helicase complex and the sliding clamp PCNA also can be visualized at sites of BrdU/EdU incorporation throughout amplification (Claycomb *et al.* 2002). Using these cell biological approaches, it has been possible to study fork elongation dynamics as well as the colocalization of other proteins and chromatin factors directly at the replication fork (Claycomb *et al.* 2002; Park *et al.* 2007; Nordman *et al.* 2014; Alexander *et al.* 2015).

Molecular biology tools, both quantitative PCR and aCGH, have permitted replication fork progression to be tracked by changes in copy number at the amplicons in DNA isolated from staged egg chambers. These approaches have allowed for high-resolution analysis of fork progression during amplification, and they have uncovered genomic sites that impede fork progression, changing the slope of the copy number gradients (Alexander *et al.* 2015). Quantitative analysis of DNA copy number has been used to examine the effects of mutations on replication fork progression.

#### Mutations that enhance fork elongation:

As discussed previously, quantification of amplification domains in *SuUR* mutants demonstrated that loss of function of this protein results in increased fork progression, with replication forks at the amplicons elongating twice as far compared to wild-type flies over the same developmental time. Thus the normal function of the SUUR protein is to impede fork progression. A *cycE* allele, *cycE^1F36^*, exhibits increased double-bar gap distances and a wider gradient of amplified DNA copy number without altering origin firing or the developmental timing of the gene amplification program (Park *et al.* 2007). This is a surprising finding, as it reveals a previously unrecognized role of Cyclin E in fork elongation, a cell cycle factor well known for its role in helicase activation during replication initiation. The replication phenotype of *cycE^1F36^* is semidominant, suggesting that the allele may be a gain-of-function mutation, promoting the progression of replication forks during amplification. However, how Cyclin E acts at the replication fork remains unclear.

#### Fork instability and DNA damage during rereplication:

**Figure 6:**
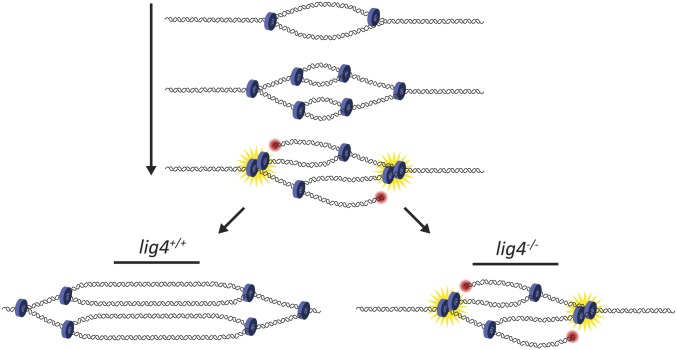
Gene amplification as a model for rereplication and DNA damage repair. The first origin firing event produces two replication forks moving in opposite directions. Origin reinitiation generates a second set of forks, such that there are now two sets of forks traveling in each direction. If the first set of forks is stalled, the second set of forks can collide with them (yellow starbursts), generating double-stranded DNA breaks (red circle). Lig4 is crucial for repairing these breaks to allow continued fork progression and replication of the domain. In the absence of Lig4, fork progression is severely hampered.

During amplification, repeated origin firing generates multiple replication forks in close proximity moving in the same direction. One possible consequence of this close arrangement of trailing forks is collision between forks. Upon collision, replication forks may collapse, resulting in DSBs in the DNA. In support of this idea, γH2Av is enriched at the amplicons specifically at the elongating replication forks, suggesting that rereplication generates a pileup of replication forks that are prone to “rear-end” collisions that may cause the formation of DSBs in the DNA (Alexander *et al.* 2015) (Figure 6). A paired-end high-throughput sequencing approach in amplification-stage egg chambers highlighted the enrichment of several deletions at the *DAFC-66D* origin, suggesting that breaks are generated and repaired in this domain (Yarosh and Spradling 2014); however, whether the observed deletions are derived from the follicle cells, nurse cells, or oocyte remains to be determined.

Interestingly, full progression of the replication forks at the amplicons requires DNA damage response signaling, as *chk1* and *chk2* mutants and a separation-of-function *mus101* allele that specifically affects DNA damage signaling function (Kondo and Perrimon 2011) exhibit significantly decreased fork progression (Alexander *et al.* 2015). These results indicate that signaling of DNA damage is critical for continued fork progression during rereplication and suggest that repair of DSBs is important for the integrity of forks moving through the region.

As many copies of the amplified region are generated during the endocycle and gene amplification, this would provide many templates from which damaged DNA in the region can be repaired by homologous recombination (HR). Surprisingly, mutants for the key HR factors BRCA2 and SpnA (a *Drosophila* homolog of Rad51) do not exhibit hampered fork progression (Alexander *et al.* 2015), and a double mutant for both homologs of Rad51, SpnA and SpnB, exhibits increased fork progression compared to wild-type controls at all amplicons (Alexander *et al.* 2016). Thus HR repair is not the main DSB repair mechanism and actually inhibits fork progression during gene amplification. Instead, a mutant for Lig4, a critical component of the nonhomologous end-joining (NHEJ) pathway, shows significantly reduced fork progression, indicating that NHEJ is a primary repair pathway utilized during gene amplification to repair DSBs and to allow subsequent forks to progress to normal levels (Alexander *et al.* 2015) (Figure 6). Finally, Mus308, a component of the microhomology-mediated end-joining pathway, allows proper fork progression at a subset of amplicons (Alexander *et al.* 2016). As amplifying follicle cells are nondividing cells whose functions are required over a short developmental time window (∼7.5 hr), it is possible that the quick repair of DNA damage offered by end-joining pathways is more advantageous during gene amplification than the homologous recombination pathway. Studies from human and yeast systems indicate that end-joining pathways like NHEJ can be completed in 30–70 min, while HR requires 5–7 hr (Rapp and Greulich 2004; Mao *et al.* 2008; Hicks *et al.* 2011).

Together, follicle cell gene amplification proves to be a powerful developmental replication system to dissect the molecular consequences of rereplication. The generation of two trailing replication forks in close proximity can result in fork collision and collapse, leading to the generation of DNA damage. If this damage is not repaired, this can pose serious consequences for subsequent forks moving through the damaged region, leading to genome instability.

## Conclusions, Implications, and Future Directions

### Differential regulation of origin activation

Research in *Drosophila* has been key in our understanding of what defines a metazoan replication origin and its activation. The ability to identify ORC binding sites in a variety of differentiated cell types has revealed a high degree of tissue specificity of origin positioning within the genome. Although ORC is enriched at promoter sites, the tissue specificity of ORC binding cannot be explained by promoter activity. A key future direction will be to decipher the chromatin configurations and chromosome conformation that designate origin and ORC positioning. The tools in *Drosophila* will permit identification of the state of chromatin modifications and associated proteins at origins and correlation with origin activity as well as contacts between origins and other chromosomal sequences. The ability to conditionally eliminate gene function will be a significant advantage in testing causality in regulation of origin activity. The ability to track the activation of specific origins during gene amplification revealed at least three distinct mechanisms of origin activation, including the possibility of ORC-independent initiation. Analyzing whether these mechanisms operate at origins during a canonical S phase and whether the other amplicon origins utilize additional activation mechanisms will be important. The follicle cells provide the opportunity to decipher how controls that normally prevent refiring of a replication origin can be overcome. Given the high frequency of gene amplification in cancer cells and the likelihood that many of these increases in copy number may result from unregulated origin activation (Hook *et al.* 2007; Beroukhim *et al.* 2010; Green *et al.* 2010; Matsui *et al.* 2013), the *Drosophila* amplicons will continue to produce relevant insights in our understanding of metazoan replication control.

### Developmental control of replication timing and fork progression

In S phase in dividing or endocycling cells, replication timing is regulated such that some genomic regions replicate early in S phase while others regulate late, a property shared between *Drosophila* and mammalian cells. Both the mechanism that dictates when origins become active and the biological significance of replication timing remain to be determined, but it is notable that replication timing profiles are relatively conserved across cell types. Recent advances in analyses of DNA replication in *Drosophila* make it an ideal model in which to define the control and role of replication timing. Replication timing profiles have been defined molecularly in cell culture and by cell biological approaches in polytene chromosomes, in which replication protein localization can be correlated with S-phase stages. The function of chromosomal proteins and chromatin modifications also can be linked to time in S phase, exploiting the extensive mutant collection in *Drosophila* and RNA interference (RNAi) tools. A crucial question to be solved is how genomic regions are established that lack ORC binding. Another is whether genomic rearrangements resulting from underreplication serve biological functions.

Both the differential replication systems in which gene copy number is decreased through underreplication and in which copy number is increased through gene amplification have permitted metazoan replication fork progression and destabilization to be visualized and analyzed. This led to the identification of the chromatin protein SUUR as a repressor of replication and inhibitor of fork progression and has uncovered links between this protein and other chromatin proteins as well as replication components. Further insights into the tissue specificity of underreplicated domains and the mechanisms of their designation will be critical to our understanding of how chromatin configuration can affect the elongation phase of DNA replication. These principles will be applicable to mammalian cells and thus to our understanding of common chromosomal fragile sites.

Both underreplication and gene amplification lead to genome instability, in the former due to replication fork instability and in the latter due to replication fork collisions. The double-strand breaks that result from these events can lead to genomic rearrangements. These models are powerful in defining repair mechanisms that can restore fork progression to prevent rearrangements, with important implications for genome stability in mammalian cells.
